# Supplementary material for: Capturing the experiences of UK healthcare workers during the COVID-19 pandemic: A structural topic modelling analysis of 7,412 free-text survey responses
Source: PLoS One. 2022 Oct 7;17(10):e0275720. doi: 10.1371/journal.pone.0275720 (PMC9543686; doi:10.1371/journal.pone.0275720)
Supplement: S1 File — (PDF) [file pone.0275720.s001.pdf]

1. Short Survey (first part)

Start of Block: Intro+consent

Q3 **Welcome to NHS CHECK** **NHS CHECK: Health & Experiences of staff working at NHS Trusts and Nightingale Hospitals** The outbreak of the COVID-19 pandemic has affected the lives of all of us working in the NHS, with many of us experiencing major disruption to our work and home lives. NHS CHECK is a major confidential study of everyone in your Trust and selected others, finding out how this pandemic is impacting on staff. Your input will help us understand how better to prepare and support our colleagues now and in the future. **NHS CHECK is open to anyone over 18 working in any role within a participating NHS Trust or Nightingale Hospital.** When you sign up to NHS CHECK you will be asked to fill in a 5-10 minute survey asking about you, your work and your health. There is also a longer survey too, if you have time. We will then get in touch with you in about 3, 12 and 18 months' time to find out how you are doing. These surveys will help us understand how things change for our colleagues over time.
Resources:

The **NHS website** at www.people.nhs.uk contains resources for supporting your mental health and also has a dedicated helpline for NHS staff affected by COVID-19. To contact the **NHS helpline**, phone 0300 131 7000, or text FRONTLINE to 85258. **Mind's** website at www.mind.org.uk has useful resources to help you cope if you are feeling anxious, worried or isolated. See **Samaritans** at www.samaritans.org if you are worried about your mental health. You can also **call the Samaritans** day or night if you need someone to talk to without judgement on 116 123. The **World Health Organization** at www.who.int has a document detailing mental health and psychosocial considerations during this outbreak.

You can also download the participant information sheet or contact the team here: nhscheck@kcl.ac.uk.

This study received ethical approval from the Health Research Authority (reference number: 282686)

---

*

Q5 I have read and understood this information about the NHS CHECK study.

◯ Yes (1)

◯ No (2)

---

*

Q7 I agree to take part in NHS CHECK and the long-term, storage of my survey data in line with GDPR.

I understand that although this involves follow up surveys, I can choose to withdraw at any time.

◯ Yes (1)

◯ No (2)

Q9 Would you like to hear about other studies in the future?

◯ Yes (1)

◯ No (2)

Q11 Please complete the below validation.

End of Block: Intro+consent

Start of Block: Follow-up

Q15
Thank you for consenting to take part in NHS CHECK. Now please register for NHS CHECK by providing us with your contact details:

We will use your contact details to send you follow-up surveys and important information that is directly connected to the NHS CHECK study.

Q17 First name (this will be used to personalise future emails to you):

____

*

Q19 Preferred work/personal email (required):

____

*

Q21 Alternative work/personal email (optional):

____

*

Q23 Mobile number (optional):

____

End of Block: Follow-up

Start of Block: Role Check

Q13 Which NHS Trust / Nightingale Hospital is your role based at? (required)

Please select all that apply:

▢ Guy’s and St. Thomas’ NHS Foundation Trust (1)

▢ King’s College Hospital (KCH) (4)

▢ Princess Royal University Hospital (PRUH) (14)

▢ South London and Maudsley NHS Foundation Trust (11)

▢ Nightingale Hospital - Bristol (5)

▢ Nightingale Hospital - Cardiff (6)

▢ Nightingale Hospital - Exeter (8)

▢ Nightingale Hospital - Leeds / Harrogate (13)

▢ Nightingale Hospital - London (7)

▢ Nightingale Hospital - Manchester (9)

▢ Other (12)

▢ Cambridgeshire and Peterborough NHS Foundation Trust (15)

▢ Norfolk and Norwich University Hospitals NHS Foundation Trust (21)

Display This Choice:

If False = True

▢ North West Anglia NHS Foundation Trust (22)

▢ Nottinghamshire Healthcare NHS Foundation Trust (23)

▢ University Hospitals of Derby and Burton NHS Foundation Trust (24)

☐ University Hospitals of Leicester NHS Trust (25)

☐ Avon and Wiltshire Mental Health Partnership NHS Trust (26)

☐ Cambridge University Hospitals NHS Foundation Trust (27)

☐ East Suffolk and North Essex NHS Foundation Trust (28)

☐ Gloucestershire Hospitals NHS Foundation Trust (29)

☐ Sheffield Health and Social Care Trust (30)

☐ Bradford Teaching Hospitals NHS Foundation Trust (31)

☐ Tees Esk and Wear Valleys NHS Foundation Trust (32)

☐ Royal Papworth Hospital NHS Foundation Trust (33)

☐ Lancashire & South Cumbria NHS Foundation Trust (34)

☐ Cornwall Partnership NHS Foundation Trust (35)

☐ Devon Partnership NHS Trust (36)

Display This Question:

If Which NHS Trust / Nightingale Hospital is your role based at? (required) Please select all that... = Other

Q15 Please specify:

____

End of Block: Role Check

Start of Block: Demographics

Q25 **First, some questions about you.**

Q27 Your age (years):

____

Q29 Gender:

- ○ Male (1)
- ○ Female (2)
- ○ Other (3)
- ○ Prefer not to say (4)

Q31 What is your relationship status?

- ○ Married / Civil partnership (100)
- ○ Co-habiting / In a relationship (105)
- ○ Divorced / Separated (102)
- ○ Single (106)
- ○ Widowed (101)

Q66
What is your ethnic group?

Please choose one option that best describes your ethnic group or background.

- ○ White English/Welsh/Scottish/Northern Irish/British (1)
- ○ White Irish (4)
- ○ White Gypsy or Irish Traveller (5)
- ○ Any other White background (6)
- ○ Mixed White and Black Caribbean (7)
- ○ Mixed White and Black African (11)
- ○ Mixed White and Asian (12)
- ○ Any other Mixed/Multiple ethnic background (13)
- ○ Indian (14)
- ○ Pakistani (19)
- ○ Bangladeshi (20)
- ○ Chinese (21)
- ○ Any other Asian background (22)
- ○ Black African (23)
- ○ Black Caribbean (26)
- ○ Any other Black background (27)
- ○ Arab (28)
- ○ Any other ethnic group (30)

Q35 What is your country of birth?

- United Kingdom (1)
- EU (not UK) (2)
- Other (please specify below) (3)

Display This Question:

If What is your country of birth? = Other (please specify below)

Q63 Please specify your country of birth:

____

Display This Question:

If What is your country of birth? = EU (not UK)

Or What is your country of birth? = Other (please specify below)

Q37 How long have you been living in the United Kingdom?

- < 1 year (2)
- 1 - 2 years (3)
- 3 - 5 years (4)
- 6 - 10 years (5)
- 11 - 20 years (6)
- 21 - 29 years (7)
- > 30 years (8)
- Prefer not to say (9)

End of Block: Demographics

Start of Block: Occupational Demographics (1 question)

Q39 What is your role? (required)

- ◯ Clinical (1)
- ◯ Non-clinical (2)
- ◯ Both (5)

Display This Question:

If What is your role? (required) = Both

Q62 If both, what is your main role? (required)

- ◯ Clinical (1)
- ◯ Non-clinical (2)

Q72 Are you a student?

- ◯ Yes (1)
- ◯ No (2)

Q73 Are you a volunteer?

- ◯ Yes (4)
- ◯ No (5)

End of Block: Occupational Demographics (1 question)

Start of Block: Occupational Demographics - Non-clinical

Display This Question:

If What is your role? (required) = Non-clinical

Or If both, what is your main role? (required) = Non-clinical

Q41 What is your occupational group? (Non-clinical)

- ○ Administrative and Clerical (1)
- ○ Catering Services (27)
- ○ Chaplaincy (28)
- ○ Clinical Support (39)
- ○ Domestic Services (29)
- ○ Estates Services (30)
- ○ Finance (31)
- ○ Healthcare Scientists (laboratory, technician) (32)
- ○ Human Resources (33)
- ○ IT Support (40)
- ○ Management (34)
- ○ Research / Academic (41)
- ○ Social Services (social worker) (38)
- ○ Support Services (driver, porter, security, stores) (42)
- ○ Other (please specify below) (25)
- ○ Prefer not to say (26)

Display This Question:

If What is your occupational group? (Non-clinical) = Other (please specify below)

Q43 Please specify your occupational group:

____

Display This Question:

If What is your role? (required) = Non-clinical

Or If both, what is your main role? (required) = Non-clinical

Q45 What is your Agenda for Change pay rate?

- ◯ Not applicable (1)
- ◯ Student (4)
- ◯ Band 1 (5)
- ◯ Band 2 (6)
- ◯ Band 3 (7)
- ◯ Band 4 (8)
- ◯ Band 5 (9)
- ◯ Band 6 (10)
- ◯ Band 7 (11)
- ◯ Band 8 (12)
- ◯ Band 9 (13)
- ◯ Don't know or prefer not to say (14)

End of Block: Occupational Demographics - Non-clinical

Start of Block: Occupational Demographics - Clinical

Display This Question:

If What is your role? (required) = Clinical

Or If both, what is your main role? (required) = Clinical

Q47 What is your occupational group? (Clinical)

- ○ Dietitian (1)
- ○ Doctor (17)
- ○ Healthcare Assistant/Nursing Assistant (18)
- ○ Healthcare Scientist (laboratory, technician) (19)
- ○ Medical Associate/Assistant Professions (20)
- ○ Midwife (21)
- ○ Nurse (22)
- ○ Occupational Therapist (23)
- ○ Paramedic/Ambulance Service (24)
- ○ Pharmacist/Pharmacy Technician (25)
- ○ Physiotherapist (26)
- ○ Psychologist/Assistant Psychologist (27)
- ○ Radiographer (28)
- ○ Speech & Language Therapist (29)
- ○ Ward Manager (14)
- ○ Other (please specify below) (15)
- ○ Prefer not to say (16)

Display This Question:

If What is your occupational group? (Clinical) = Other (please specify below)

Q49 Please specify your occupational group:

____

Display This Question:

If What is your occupational group? (Clinical) = Doctor

Q51 Which of the following describes your subgroup?

- ○ Interim F1 (10)
- ○ In training (F1/F2) (1)
- ○ In training (Core Trainee) (2)
- ○ In training (Specialty Registrar) (3)
- ○ In training (GP Trainee) (4)
- ○ Specialty Doctor (5)
- ○ Consultant (6)
- ○ GP (7)
- ○ Other (9)

Display This Question:

If What is your occupational group? (Clinical) = Dietitian

Or What is your occupational group? (Clinical) = Healthcare Assistant/Nursing Assistant

Or What is your occupational group? (Clinical) = Healthcare Scientist (laboratory, technician)

Or What is your occupational group? (Clinical) = Medical Associate/Assistant Professions

Or What is your occupational group? (Clinical) = Midwife

Or What is your occupational group? (Clinical) = Nurse

Or What is your occupational group? (Clinical) = Occupational Therapist

Or What is your occupational group? (Clinical) = Paramedic/Ambulance Service

Or What is your occupational group? (Clinical) = Pharmacist/Pharmacy Technician

Or What is your occupational group? (Clinical) = Physiotherapist

Or What is your occupational group? (Clinical) = Psychologist/Assistant Psychologist

Or What is your occupational group? (Clinical) = Radiographer

Or What is your occupational group? (Clinical) = Ward Manager

Or What is your occupational group? (Clinical) = Other (please specify below)

Q53 What is your Agenda for Change pay rate?

- Not applicable (1)
- Student (4)
- Band 1 (5)
- Band 2 (6)
- Band 3 (7)
- Band 4 (8)
- Band 5 (9)
- Band 6 (10)
- Band 7 (11)
- Band 8 (12)
- Band 9 (13)
- Don't know or prefer not to say (14)

End of Block: Occupational Demographics - Clinical

Start of Block: Occupational Demographics - All

Q55 Are you a permanent staff member?

- Yes (1)
- Fixed-term contract (2)
- Training contract (3)
- Locum/Bank/Agency/Shift/Contractor (4)
- Other (5)

Q57 Do you line manage or directly supervise other staff members?

- Yes (1)
- Yes - but only since COVID-19 (coronavirus) pandemic (2)
- No (3)
- Unsure (4)

Q59 Length of time since gaining professional registration

- < 1 year (2)
- 1 - 2 years (3)
- 3 - 5 years (4)
- 6 - 10 years (5)
- 11 - 20 years (6)
- 21 - 29 years (7)
- > 30 years (9)
- < 3 months (1)

Display This Question:

If Length of time since gaining professional registration = < 1 year

Q61 Were you fast-tracked to employment due to COVID-19 (coronavirus)?

- Yes (1)
- No (2)

Display This Question:

If Were you fast-tracked to employment due to COVID-19 (coronavirus)? = Yes

Q69 Are you working (paid or voluntary) in the same location / teams as previous placements you may have undertaken?

- Yes (1)
- No (2)
- Not applicable (3)

Display This Question:

If Were you fast-tracked to employment due to COVID-19 (coronavirus)? = Yes

Q70 How do you feel about starting your new role?

- Very positive (1)
- Positive (2)
- Neutral (3)
- Negative (4)
- Very negative (5)

Display This Question:

If Were you fast-tracked to employment due to COVID-19 (coronavirus)? = Yes

Q67 How well do you feel prepared for your new role?

- Not at all (1)
- A little bit (2)
- Moderately (3)
- Quite a bit (4)
- Extremely (5)

Display This Question:

If Were you fast-tracked to employment due to COVID-19 (coronavirus)? = Yes

Q68 Do you feel the organisation where you are working (paid or voluntary) is supporting you in the transition from student to qualified professional?

- Not at all (1)
- A little bit (2)
- Moderately (3)
- Quite a bit (4)
- Extremely (5)

Display This Question:

If Length of time since gaining professional registration = 11 - 20 years

Or Length of time since gaining professional registration = 21 - 29 years

Or Length of time since gaining professional registration = > 30 years

Q63 Did you return from retirement to employment due to COVID-19 (coronavirus)?

- Yes (1)
- No (2)

Display This Question:

If Which NHS Trust / Nightingale Hospital is your role based at? (required) Please select all that... = Guy's and St. Thomas' NHS Foundation Trust

Or Which NHS Trust / Nightingale Hospital is your role based at? (required) Please select all that... = King's College Hospital (KCH)

Or Which NHS Trust / Nightingale Hospital is your role based at? (required) Please select all that... = Princess Royal University Hospital (PRUH)

Or Which NHS Trust / Nightingale Hospital is your role based at? (required) Please select all that... = South London and Maudsley NHS Foundation Trust

Or Which NHS Trust / Nightingale Hospital is your role based at? (required) Please select all that... = Other

Q65 Length of time at **current** employing organisation:

- ◯ < 3 months (1)
- ◯ 3 - 12 months (2)
- ◯ 1 - 2 years (3)
- ◯ 3 - 5 years (4)
- ◯ 6 - 10 years (5)
- ◯ 11 - 19 years (6)
- ◯ 20 - 29 years (7)
- ◯ > 30 years (8)

Display This Question:

If Which NHS Trust / Nightingale Hospital is your role based at? (required) Please select all that... = Cambridgeshire and Peterborough NHS Foundation Trust

Or Which NHS Trust / Nightingale Hospital is your role based at? (required) Please select all that... = Norfolk and Norwich University Hospitals NHS Foundation Trust

Or Which NHS Trust / Nightingale Hospital is your role based at? (required) Please select all that... = North West Anglia NHS Foundation Trust

Or Which NHS Trust / Nightingale Hospital is your role based at? (required) Please select all that... = Nottinghamshire Healthcare NHS Foundation Trust

Or Which NHS Trust / Nightingale Hospital is your role based at? (required) Please select all that... = University Hospitals of Derby and Burton NHS Foundation Trust

Or Which NHS Trust / Nightingale Hospital is your role based at? (required) Please select all that... = University Hospitals of Leicester NHS Trust

Q74 Length of time at **current** employing organisation:

- ○ < 3 months (1)
- ○ 3 - 12 months (2)
- ○ 1 - 2 years (3)
- ○ 3 - 5 years (4)
- ○ 6 - 10 years (5)
- ○ 11 - 19 years (6)
- ○ 20 - 29 years (7)
- ○ > 30 years (8)

Display This Question:

If Which NHS Trust / Nightingale Hospital is your role based at? (required) Please select all that... = Avon and Wiltshire Mental Health Partnership NHS Trust

Or Which NHS Trust / Nightingale Hospital is your role based at? (required) Please select all that... = Cambridge University Hospitals NHS Foundation Trust

Or Which NHS Trust / Nightingale Hospital is your role based at? (required) Please select all that... = East Suffolk and North Essex NHS Foundation Trust

Or Which NHS Trust / Nightingale Hospital is your role based at? (required) Please select all that... = Gloucestershire Hospitals NHS Foundation Trust

Or Which NHS Trust / Nightingale Hospital is your role based at? (required) Please select all that... = Lancashire & South Cumbria NHS Foundation Trust

Or Which NHS Trust / Nightingale Hospital is your role based at? (required) Please select all that... = Cornwall Partnership NHS Foundation Trust

Or Which NHS Trust / Nightingale Hospital is your role based at? (required) Please select all that... = Devon Partnership NHS Trust

Q75 Length of time at **current** employing organisation:

- ◯ < 3 months (1)
- ◯ 3 - 12 months (2)
- ◯ 1 - 2 years (3)
- ◯ 3 - 5 years (4)
- ◯ 6 - 10 years (5)
- ◯ 11 - 19 years (6)
- ◯ 20 - 29 years (7)
- ◯ > 30 years (8)

Display This Question:

If Which NHS Trust / Nightingale Hospital is your role based at? (required) Please select all that... = Sheffield Health and Social Care Trust

Or Which NHS Trust / Nightingale Hospital is your role based at? (required) Please select all that... = Bradford Teaching Hospitals NHS Foundation Trust

Or Which NHS Trust / Nightingale Hospital is your role based at? (required) Please select all that... = Tees Esk and Wear Valleys NHS Foundation Trust

Or Which NHS Trust / Nightingale Hospital is your role based at? (required) Please select all that... = Royal Papworth Hospital NHS Foundation Trust

Q76 Length of time at **current** employing organisation:

- < 3 months (1)
- 3 - 12 months (2)
- 1 - 2 years (3)
- 3 - 5 years (4)
- 6 - 10 years (5)
- 11 - 19 years (6)
- 20 - 29 years (7)
- > 30 years (8)

Display This Question:

If Which NHS Trust / Nightingale Hospital is your role based at? (required) Please select all that... = Nightingale Hospital - Bristol

Or Which NHS Trust / Nightingale Hospital is your role based at? (required) Please select all that... = Nightingale Hospital - Cardiff

Or Which NHS Trust / Nightingale Hospital is your role based at? (required) Please select all that... = Nightingale Hospital - Exeter

Or Which NHS Trust / Nightingale Hospital is your role based at? (required) Please select all that... = Nightingale Hospital - Leeds / Harrogate

Or Which NHS Trust / Nightingale Hospital is your role based at? (required) Please select all that... = Nightingale Hospital - London

Or Which NHS Trust / Nightingale Hospital is your role based at? (required) Please select all that... = Nightingale Hospital - Manchester

Q67 Length of time working at Nightingale Hospital:

- ○ < 1 month (1)
- ○ < 2 months (2)
- ○ < 3 months (3)
- ○ > 3 months (4)

Q69 Prior to the COVID-19 (coronavirus) pandemic did you work in a different setting?

- ○ Yes (1)
- ○ No (2)

Display This Question:

If Prior to the COVID-19 (coronavirus) pandemic did you work in a different setting? = Yes

Q71 What setting did you **usually** work in before the COVID-19 (coronavirus) pandemic?

- ○ Anaesthetics (4)
- ○ ICU/Critical Care (9)
- ○ Medicine (including sub-specialities) (6)
- ○ Ophthalmology (7)
- ○ Paediatrics (10)
- ○ Palliative Care (11)
- ○ Primary Care (12)
- ○ Psychiatry - Community (16)
- ○ Psychiatry - Inpatient/Forensic (13)
- ○ Psychiatry - Liaison (14)
- ○ Radiology (21)
- ○ Secure Setting (locked wards, prisons) (17)
- ○ Surgery (including sub-specialities) (19)
- ○ Women's Health (20)
- ○ Hospital (other) (18)
- ○ Other (please specify below) (3)
- ○ Accident and Emergency (23)

Display This Question:

If What setting did you usually work in before the COVID-19 (coronavirus) pandemic? = Other (please specify below)

Q65 Please specify the setting you usually worked in:

____

Q77 What setting are you **currently** working in?

- ◯ Anaesthetics (1)
- ◯ ICU/Critical Care (9)
- ◯ Medicine (including sub-specialities) (4)
- ◯ Ophtalmology (5)
- ◯ Paediatrics (6)
- ◯ Palliative Care (12)
- ◯ Primary Care (13)
- ◯ Psychiatry - Community (10)
- ◯ Psychiatry - Inpatient/Forensic (14)
- ◯ Psychiatry - Liaison (15)
- ◯ Radiology (16)
- ◯ Secure Setting (locked wards, prisons) (18)
- ◯ Surgery (including sub-specialities) (17)
- ◯ Women's Health (11)
- ◯ Hospital (other) (8)
- ◯ Other (please specify below) (3)
- ◯ Accident and Emergency (20)

Display This Question:

If What setting are you currently working in? = Other (please specify below)

Q64 Please specify the setting you are currently working in:

____

Display This Question:

If Prior to the COVID-19 (coronavirus) pandemic did you work in a different setting? = Yes

Q75 How do you feel about having been or being redeployed to a new role?

- ◯ Very positive (1)
- ◯ Positive (2)
- ◯ Neutral (3)
- ◯ Negative (4)
- ◯ Very Negative (5)

End of Block: Occupational Demographics - All

Start of Block: COVID-19 & Work

Q79 We would like to know a bit more about your recent work experiences.

As part of your work have you been in contact with people with suspected COVID-19 (coronavirus) symptoms?

- ◯ Yes (1)
- ◯ No (2)
- ◯ Unsure (4)

Q81 Further,

	Not at all (1)	A little bit (2)	Moderately (3)	Quite a bit (4)	Extremely (5)
Do you feel unsafe or vulnerable to being infected/reinfected with COVID-19 (coronavirus) at work? (9)	○	○	○	○	○
Are you satisfied that there are clear infection control policies at work? (10)	○	○	○	○	○

Display This Question:

If What is your role? (required) = Clinical

Q83 How often do you currently perform aerosolising procedures?

○ Never (29)

○ Rarely (30)

○ Sometimes (31)

○ Often (32)

○ Most/all patient contact (33)

Q85 Do you have access to adequate personal protective equipment (PPE)?

- Never (36)
- Sometimes (35)
- Often (34)
- Always (33)
- Not applicable (38)
- Prefer not to answer (37)

End of Block: COVID-19 & Work

Start of Block: Support + NHS support programmes x1 question

Q87 Since the COVID-19 (coronavirus) pandemic

	Not at all (1)	A little bit (2)	Moderately (3)	Quite a bit (4)	Extremely (5)
How well do you feel supported by your colleagues? (1)	○	○	○	○	○
How well do you feel supported by your supervisors/managers? (2)	○	○	○	○	○
How well do you feel supported by your family and/or friends? (3)	○	○	○	○	○

Q115 We would like to know if you have accessed any staff support programmes.

Please select any of the following you have used:

- ☐ Relaxation / time out areas (2)
- ☐ Helplines (3)
- ☐ Employee Assistance Programmes (8)
- ☐ Wellbeing activities (e.g. mindfulness, relaxation etc.) (4)
- ☐ Other (please specify below) (5)
- ☐ Did not use any (7)
- ☐ Not Applicable (6)

Display This Question:

If We would like to know if you have accessed any staff support programmes. Please select any of the... = Other (please specify below)

Q117 Please specify other staff support programmes you have accessed:

____

End of Block: Support + NHS support programmes x1 question

Start of Block: COVID-19 Individual Experiences

Q107 Do you, or have you had, symptoms that you suspect might be the result of a COVID-19 (coronavirus) infection?

- ○ Yes (15)
- ○ No (17)
- ○ Unsure (16)

Q109 Have you been tested for COVID-19 (coronavirus)?

- ◯ Yes (positive) (5)
- ◯ Yes, tested negative (7)
- ◯ Yes, awaiting result (8)
- ◯ Yes but inconclusive (10)
- ◯ Not tested (9)

Q111 Are you isolating, or have you had to isolate due to having COVID-19 (coronavirus) symptoms?

- ◯ Yes (5)
- ◯ No (6)

Q113 Are you, or have you had to quarantine due to other people in your household having COVID-19 (coronavirus) symptoms?

- ◯ Yes (4)
- ◯ No (5)

End of Block: COVID-19 Individual Experiences

Start of Block: General Health

Q105
Have you been diagnosed by a doctor or other health professional with any of the following **in the last 12 months**?

Please select all that apply:

- ▢ Anxiety (1)
- ▢ Asthma/COPD (4)
- ▢ Cancer (5)
- ▢ Chronic pain (6)
- ▢ Depression (7)
- ▢ Diabetes (8)
- ▢ Heart attack / Heart disease (9)
- ▢ High blood pressure (10)
- ▢ Post-traumatic stress disorder (11)
- ▢ Stroke (12)
- ▢ None of the above (13)

Q89 These questions ask about your general health.

Within the **last few weeks**, how often have you

	Better than usual (1)	Same as usual (2)	Worse than usual (3)	Much worse than usual (4)
Been able to concentrate on whatever you're doing? (1)	○	○	○	○

Q91 or

	Not at all (1)	No more than usual (2)	Rather more than usual (3)	Much more than usual (4)
Lost much sleep over worry? (1)	○	○	○	○

Q93 or

	More so than usual (1)	Same as usual (2)	Less useful than usual (3)	Much less than usual (4)
Felt that you were playing a useful part in things? (1)	○	○	○	○
Felt capable of making decisions about things? (13)	○	○	○	○

Q95 or

	Not at all (1)	No more than usual (2)	Rather more than usual (3)	Much more than usual (4)
Felt constantly under strain? (1)	○	○	○	○
Felt that you couldn’t overcome your difficulties? (13)	○	○	○	○

Q97 or

	More so than usual (1)	Same as usual (2)	Less so than usual (3)	Much less than usual (4)
Been able to enjoy your normal day-to-day activities? (1)	○	○	○	○

Q99 Within the **last few weeks**, how often have you

	Not at all (1)	Same as usual (2)	Less able than usual (3)	Much less able (4)
Been able to face up to your problems? (1)	○	○	○	○

Q101 or

	Not at all (1)	No more than usual (2)	Rather more than usual (3)	Much more than usual (4)
Been feeling unhappy or depressed? (1)	○	○	○	○
Been losing confidence in yourself? (13)	○	○	○	○
Been thinking of yourself as a worthless person? (14)	○	○	○	○

Q103 or

	More so than usual (1)	About the same as usual (2)	Less so than usual (3)	Much less than usual (4)
Been feeling reasonably happy all things considered? (1)	○	○	○	○

End of Block: General Health

Start of Block: END short survey

Q119 Is there anything else you think we should know about your experiences of the COVID-19 (coronavirus) pandemic?

____

Q121

We would like to ask you a few more questions. Your help in answering further questions will be of great support in helping us understand how better to prepare and support our colleagues now and in the future.

This will take a further 20-30 minutes to complete. We are grateful for the time you can spare on this.

Please indicate your preference:

○ Yes, happy to proceed. You will now be re-directed to the second part of the survey. You may see a screen with your survey ID. Please click the arrow below and proceed. (1)

○ Maybe later, happy for you to send me a reminder in a couple of days. (2)

○ No (4)

End of Block: END short survey

2. Long Survey (second part)

Start of Block: Experience at work - second part

Q3 Thank you for your continued participation.

Survey ID: ${e://Field/Login%20ID}

Q5 Please complete the below validation: (required)

Q13
We would like to know a little bit more about your experience at work.

Are you working part-time or full-time?

○ Part-time (1)

○ Full-time (2)

Q11 In the past 2 weeks...

	Never (1)	Rarely (2)	Sometimes (3)	Often (4)	Always (5)	Not Applicable (6)
I have had to do work I do not feel sufficiently competent or trained to do. (1)	○	○	○	○	○	○
I or my team have had to provide significantly worse care than usual to our patients or deny them treatment that would normally be available. (3)	○	○	○	○	○	○
I have felt let down because I am working with insufficient staffing or resources. (8)	○	○	○	○	○	○

Q7 We would like to know a little bit more about your team.

	Not at all (1)	A little bit (2)	Moderately (3)	Quite a bit (4)	Extremely (5)
Do you feel part of a team? (6)	o	o	o	o	o
I feel a sense of comradeship (or closeness) between myself and other people in the team. (10)	o	o	o	o	o

Q9 Recently,

	Very negative change (2)	Negative change (3)	No change (4)	Positive change (5)	Very positive change (6)
how have relationships within your team changed since the COVID-19 (coronavirus) pandemic? (If in a new team compare to team worked in prior to the pandemic) (1)	o	o	o	o	o

Q15 In the past **4 weeks**, how many days of work did you miss for health reasons?

- ○ 0 (1)
- ○ 1 - 2 (2)
- ○ 3 - 7 (3)
- ○ 8 - 14 (4)
- ○ 15 - 21 (5)
- ○ 22+ (6)
- ○ Prefer not to say (7)

Q17 If you were concerned about unsafe clinical practice, would you know how to report it?

- ○ Yes (1)
- ○ No (2)

Display This Question:

If If you were concerned about unsafe clinical practice, would you know how to report it? = Yes

Q19 Raising concerns about unsafe clinical practice:

	Strongly disagree (1)	Disagree (2)	Neither agree nor disagree (3)	Agree (4)	Strongly agree (5)
I would feel secure raising concerns about unsafe clinical practice. (1)	○	○	○	○	○
I am confident that my organisation would address my concern. (2)	○	○	○	○	○

End of Block: Experience at work - second part

Start of Block: Support - Second part

Q12 Within the last few weeks:

	Not at all (1)	A little bit (2)	Moderately (3)	Quite a bit (4)	Extremely (5)
Do you feel more isolated from family/friends because of your work role? (1)	o	o	o	o	o
Do you feel like friends, family or strangers are treating you differently **(positively)** because you work in a hospital/care setting? (2)	o	o	o	o	o
Do you feel like friends, family or strangers are treating you differently **(negatively)** because you work in a hospital/care setting? (3)	o	o	o	o	o

Q14 We would like to know if you have accessed any staff support programmes.

Please tick any of the following you **found helpful**:

- ☐ Relaxation / time out areas (2)
- ☐ Helplines (3)
- ☐ Employee Assistance Programmes (8)
- ☐ Wellbeing activities (e.g. mindfulness, relaxation etc.) (4)
- ☐ Other (please specify below) (5)
- ☐ Did not use (7)
- ☐ Not Applicable (6)

Display This Question:

If We would like to know if you have accessed any staff support programmes.   Please tick any of the... = Other (please specify below)

Q16 Please specify any other staff support programmes you found helpful:

____

Q18 Please select any of the following you **did not** find helpful:

- ▢ Relaxation / time out areas (2)
- ▢ Helplines (3)
- ▢ Employee Assistance Programmes (8)
- ▢ Wellbeing activities (e.g. mindfulness, relaxation etc.) (4)
- ▢ Other (please specify below) (5)
- ▢ Did not use (7)
- ▢ Not Applicable (6)

Display This Question:

If Please select any of the following you did not find helpful: = Other (please specify below)

Q20 Please specify any other staff support programmes you did not find helpful:

____

End of Block: Support - Second part

Start of Block: COVID-19 Impact on Life - Second part

Q22
The following questions relate to some possible impacts that the COVID-19 (coronavirus) pandemic may have had on your life.

In the **last 2 weeks** have you been worried about:

	Not applicable (48)	Not at all worried (49)	Not too worried (50)	Somewhat worried (51)	Very worried (52)	Extremely worried (53)
The employment status of other key earners in your household? (19)	○	○	○	○	○	○
Your household finances? (22)	○	○	○	○	○	○
The mental health and wellbeing of close family and friends? (23)	○	○	○	○	○	○
Shortage of essential supplies (food, household, medication)? (35)	○	○	○	○	○	○

Q24 We would like to know how much confidence you have in the following institutions:

	None (48)	Little (49)	Some (50)	Much (51)	A great deal (52)
How much confidence do you have in the **government** that they can handle COVID-19 (coronavirus) well? (4)	○	○	○	○	○
How much confidence do you have in the **health service** to handle COVID-19 (coronavirus) well? (5)	○	○	○	○	○
How much confidence do you have in your **organisation** to handle COVID-19 (coronavirus) well? (6)	○	○	○	○	○

End of Block: COVID-19 Impact on Life - Second part

Start of Block: COVID-19 Attitudes - Second part

Q26 The following questions relate to your responses to COVID-19 (coronavirus):

	Strongly disagree (1)	Disagree (2)	Neither agree nor disagree (3)	Agree (4)	Strongly agree (5)
I am coping well with the COVID-19 (coronavirus) pandemic. (1)	○	○	○	○	○

Q30 Has the COVID-19 (coronavirus) pandemic led to any positive changes **in your life**?

○ Yes, a lot (1)

○ Yes, some (2)

○ No (3)

Q32 Has the COVID-19 (coronavirus) pandemic led to any positive changes **at work**?

○ Yes, a lot (1)

○ Yes, some (2)

○ No (3)

End of Block: COVID-19 Attitudes - Second part

Start of Block: COVID-19 indvidual exp/caring demands - Second part

Q34
We would like to know a bit more about your personal experiences with COVID-19 (coronavirus).

Do you consider yourself as being in an at-risk group (e.g. elderly, existing health conditions or pregnant) for the COVID-19 (coronavirus)?

- ◯ Yes (1)
- ◯ No (2)

Display This Question:

If We would like to know a bit more about your personal experiences with COVID-19 (coronavirus). Do... = Yes

Q36 If yes, for what reason? Please select all that apply:

- ▢ Elderly (1)
- ▢ Existing health condition (2)
- ▢ Pregnant (3)
- ▢ Other (4)

Q51 Are you currently taking daily vitamin D supplements?

- ◯ Yes, I've bought my own Vitamin D supplements to take (1)
- ◯ Yes, I've been given Vitamin D supplements by my Trust/Nightingale Hospital (4)
- ◯ No, but I have been offered Vitamin D supplements by my Trust/Nightingale Hospital (5)
- ◯ No, and I haven't been offered Vitamin D supplements by my Trust/Nightingale Hospital (6)

Q38 Is there anybody else in your household who you consider to be in an at-risk group (e.g. elderly, existing health conditions or pregnant) for the COVID-19 (coronavirus)?

◯ Yes (1)

◯ No (2)

Q40 Do you have any children under the age of 18?

◯ Yes (1)

◯ No (2)

Display This Question:

If Do you have any children under the age of 18? = Yes

Q42 Have school closures meant you needed to adopt alternative approaches to childcare compared to usual?

◯ No (1)

◯ Yes, but NOT impacted on ability to work (2)

◯ Yes, impacted on ability to work (3)

Q44 Do you have any other dependents or caring responsibilities?

◯ Yes (1)

◯ No (2)

Q46 Is your partner also a key worker according to the Government's definition?

- Yes (1)
- No (2)
- Unsure (3)
- Not Applicable (4)

Q48
Outside of work, are you or have you been caring for someone with COVID-19 (coronavirus)?

- Yes (1)
- No (2)

Q50
Outside of work, has someone you know died from COVID-19 (coronavirus)?

- Yes (1)
- No (2)

Q52 Have any of your **colleagues/friends at work** died from COVID-19 (coronavirus)?

- Yes (1)
- No (2)

End of Block: COVID-19 indvidual exp/caring demands - Second part

---

Start of Block: Well-being (WEMWBS) - Second part

Q52
Below are some statements about feelings and thoughts.

Please tick the box that best describes your experience of each **over the last 2 weeks:**

	None of the time (1)	Rarely (2)	Some of the time (3)	Often (4)	All of the time (5)
I've been feeling optimistic about the future (1)	○	○	○	○	○
I've been feeling useful (2)	○	○	○	○	○
I've been feeling relaxed (3)	○	○	○	○	○
I've been feeling interested in other people (4)	○	○	○	○	○
I've had energy to spare (5)	○	○	○	○	○
I've been dealing with problems well (6)	○	○	○	○	○
I've been thinking clearly (7)	○	○	○	○	○
I've been feeling good about myself (8)	○	○	○	○	○
I've been feeling close to other people (9)	○	○	○	○	○
I've been feeling confident (10)	○	○	○	○	○

I’ve been able to make up my own mind about things (11)	○	○	○	○	○
I've been feeling loved (12)	○	○	○	○	○
I've been interested in new things (13)	○	○	○	○	○
I've been feeling cheerful (14)	○	○	○	○	○

End of Block: Well-being (WEMWBS) - Second part

Start of Block: Chronic Fatigue - Second Part

Q54
Please use the following scale to answer the question below:

	0 = Not at all (1)	1 (8)	2 (2)	3 (3)	4 (4)	5 (5)	6 (6)	7 (7)	8 (9)	9 (10)	10 = Extremely (11)
I am emotionally and physically exhausted (1)	○	○	○	○	○	○	○	○	○	○	○

Q56 Is the fatigue you are experiencing significantly greater than the tiredness you usually feel?

- Yes (1)
- No (2)

Q58 Does the fatigue you are experiencing interfere with your ability to do things?

- Yes (1)
- No (2)

End of Block: Chronic Fatigue - Second Part

Start of Block: Mental Health - Second Part

Q60 Over **the last 2 weeks**, how often have you been bothered by any of the following problems:

	Not at all (33)	Several days (34)	More than half the days (35)	Nearly every day (36)
Feeling nervous, anxious or on edge? (4)	○	○	○	○
Not being able to stop or control worrying? (5)	○	○	○	○
Worrying too much about different things? (6)	○	○	○	○
Had trouble relaxing? (7)	○	○	○	○
Being so restless that it is hard to sit still? (8)	○	○	○	○
Becoming easily annoyed or irritable? (9)	○	○	○	○
Feeling afraid as if something awful might happen? (10)	○	○	○	○

Q62 Over **the last 2 weeks**, how often have you been bothered by any of the following problems:

	Not at all (29)	Several days (30)	More than half the days (31)	Nearly every day (32)
Felt little interest or pleasure in doing things? (4)	○	○	○	○
Felt down, depressed or hopeless? (5)	○	○	○	○
Trouble falling or staying asleep, or sleeping too much? (6)	○	○	○	○
Felt tired or had little energy? (7)	○	○	○	○
Poor appetite or overeating? (8)	○	○	○	○
Feeling bad about yourself or that you have let yourself or your family down? (9)	○	○	○	○
Trouble concentrating on things, such as reading the newspaper or watching television (10)	○	○	○	○
Moving or speaking so slowly, that people could have noticed? Or the opposite, being so fidgety or restless that you have been moving around a lot more than usual? (11)	○	○	○	○

Thoughts that you would be better of dead or hurting yourself in some way? (12)	○	○	○	○

Q64 There may be times in everyone's life when they become very miserable and depressed and may feel like taking drastic action because of these feelings.

	Yes, in the past 2 months (31)	Yes, but not in the past 2 months (32)	No (33)
Have you ever thought of taking your life, even if you would not really do it? (4)	○	○	○
Have you ever made an attempt to take your life, by taking an overdose of tablets or in some other way? (5)	○	○	○
Have you ever harmed yourself in any way but not with the intention of killing yourself? (6)	○	○	○

Q66 Your safety:

We appreciate that you are willing to share your experiences and feelings with the research team. However, we cannot respond to information provided by you about your physical and mental health during this survey.

If you would like emotional support, or completing the survey has caused distress, we encourage you to reach out to someone you trust.

Suggestions of outside support can be found in our Participant Information Sheet and in our FAQ

End of Block: Mental Health - Second Part

Start of Block: Alcohol - Second Part

Q68 Next we would like to ask you about alcohol.

Your answers will remain confidential so please be honest. Over the last 2 months…

Q70 How often do you have a drink containing alcohol?

- ○ Never (38)
- ○ Monthly or less (39)
- ○ 2-4 times a month (40)
- ○ 2-3 times a week (41)
- ○ 4 or more times a week (42)

Skip To: End of Block If How often do you have a drink containing alcohol? = Never

---

Q72 How many drinks containing alcohol do you have on a typical day when you are drinking?

- 1 or 2 (38)
- 3 or 4 (39)
- 5 or 6 (40)
- 7 to 9 (41)
- 10 or more (42)

Q74 Please respond to the following questions:

	Never (34)	Less than monthly (35)	Monthly (36)	Weekly (37)	Daily or almost daily (38)
How often have you had 6 or more drinks on a single occasion during the last 2 months? (4)	○	○	○	○	○
How often during the last 2 months have you found that you were not able to stop drinking once you had started? (5)	○	○	○	○	○
How often during the last 2 months have you failed to do what was normally expected from you because of drinking? (6)	○	○	○	○	○
How often during the last 2 months have you needed a first drink in the morning to get yourself going after a heavy drinking session? (7)	○	○	○	○	○

How often during the 2 months have you had a feeling of guilt or remorse after drinking? (8)	○	○	○	○	○
How often during the last 2 months have you been unable to remember what happened the night before because you had been drinking? (9)	○	○	○	○	○

Q76 Have you or someone else been injured as a result of your drinking in the last 2 months?

○ Yes (23)

○ No (25)

Q78 Has a relative or friend or a doctor or another health worker been concerned about your drinking or suggested you cut down in the last 2 months?

○ Yes (31)

○ No (33)

Q80 Over the past week have you drunk more alcohol than you would usually before the COVID-19 (coronavirus) pandemic?

- ○ Less than usual (15)
- ○ About the same (16)
- ○ More than usual (17)

End of Block: Alcohol - Second Part

Start of Block: Burnout (BAT-12) - Second Part

Q82 The following statements are related to your work situation and how you experience this situation.

Please state how often each statement applies to you:

	Never (1)	Rarely (2)	Sometimes (3)	Often (4)	Always (5)
At work, I feel mentally exhausted (1)	○	○	○	○	○
After a day at work, I find it hard to recover my energy (4)	○	○	○	○	○
At work, I feel physically exhausted (5)	○	○	○	○	○
I struggle to find any enthusiasm for my work (6)	○	○	○	○	○
I feel a strong aversion towards my job (14)	○	○	○	○	○
I’m cynical about what my work means to others (15)	○	○	○	○	○
At work, I have trouble staying focused (9)	○	○	○	○	○
When I’m working, I have trouble concentrating (16)	○	○	○	○	○
I make mistakes in my work because I have my mind on other things (17)	○	○	○	○	○

At work, I feel unable to control my emotions (12)	○	○	○	○	○
I do not recognise myself in the way I react emotionally at work (18)	○	○	○	○	○
At work I may overreact unintentionally (19)	○	○	○	○	○

End of Block: Burnout (BAT-12) - Second Part

Start of Block: PTSD (PCL-6) Second Part

Q48 Over **the past 2 weeks**, how much have you been bothered by these problems:

	Not at all (41)	A little bit (42)	Moderately (43)	Quite a bit (44)	Extremely (45)
Repeated, disturbing memories, thoughts of images related to the current pandemic? (4)	○	○	○	○	○
Feeling very upset when something reminded you of the current pandemic? (5)	○	○	○	○	○
Avoiding activities or situations because they reminded you of the pandemic (this does not include activities or situations that are currently restricted or advised against)? (6)	○	○	○	○	○
Feeling distant or cut off from other people during the pandemic? (7)	○	○	○	○	○
Feeling irritable or having angry outbursts since the pandemic? (8)	○	○	○	○	○

Difficulty concentrating since the pandemic? (9)	○	○	○	○	○

End of Block: PTSD (PCL-6) Second Part

Start of Block: Moral Injury (MIES) - Second part

Q50 Please select how much you agree or disagree with each of the following statements regarding your experiences at any time since working for/with the NHS during COVID-19 (coronavirus) pandemic.

Display This Choice:

If False = True

Display This Choice:

If False = True

	Strongly Agree (1)	Moderately Agree (2)	Slightly Agree (3)	Slightly Disagree (4)	Moderately Disagree (5)	Strongly Disagree (6)
I saw things that were morally wrong (1)	○	○	○	○	○	○
I am troubled by having witnessed others' immoral acts (10)	○	○	○	○	○	○
I acted in ways that violated my own moral code or values (11)	○	○	○	○	○	○
I am troubled by having acted in ways that violated my own morals or values (12)	○	○	○	○	○	○
I violated my own morals by failing to do something that I felt I should have done (13)	○	○	○	○	○	○
I am troubled because I violated my morals by failing to do something that I felt I should have done (14)	○	○	○	○	○	○
I feel betrayed by my supervisors/managers who I once trusted (15)	○	○	○	○	○	○
I feel betrayed by co-workers who I once trusted (16)	○	○	○	○	○	○
I feel betrayed by others outside the health service who I once trusted (17)	○	○	○	○	○	○

Display This Choice: If False = True I trust my supervisors/managers and co-workers to always live up to their core values (18)	○	○	○	○	○	○
Display This Choice: If False = True I trust myself to always live up to my own moral code (19)	○	○	○	○	○	○

End of Block: Moral Injury (MIES) - Second part

Start of Block: Resilience (BRS) - Second Part

Q52 How much do you agree with the following about your life as an adult:

	Strongly disagree (27)	Disagree (28)	Neutral (29)	Agree (30)	Strongly Agree (31)
I tend to bounce back quickly after hard times (32)	○	○	○	○	○
I have a hard time making it through stressful events (33)	○	○	○	○	○
It does not take me long to recover from a stressful event (34)	○	○	○	○	○
It is hard for me to snap back when something bad happens (35)	○	○	○	○	○
I usually come through difficult times with little trouble (36)	○	○	○	○	○
I tend to take a long time to get over setbacks in my life (37)	○	○	○	○	○

End of Block: Resilience (BRS) - Second Part
